# LaDEP: A large database of English pseudo-compounds

**DOI:** 10.3758/s13428-023-02170-w

**Published:** 2023-07-18

**Authors:** Leah Auch, Karen Pérez Cruz, Christina L. Gagné, Thomas L. Spalding

**Affiliations:** 1 Department of Communication Sciences and Disorders, University of Alberta, Corbett Hall, Edmonton, AB T6G 2G4 Canada; 2 Department of Counselling Psychology, Yorkville University, Fredericton, NB E3C 2R9 Canada; 3 Department of Psychology, University of Alberta, P-217 Biological Sciences Building, Edmonton, AB T6G 2E9 Canada

**Keywords:** Pseudo-compound words, Pseudo-affixed words, Morphology, Psycholinguistics, Multimorphemic words

## Abstract

The Large Database of English Pseudo-compounds (LaDEP) contains nearly 7500 English words which mimic, but do not truly possess, a compound morphemic structure. These pseudo-compounds can be parsed into two free morpheme constituents (e.g., *car-pet*), but neither constituent functions as a morpheme within the overall word structure. The items were manually coded as pseudo-compounds, further coded for features related to their morphological structure (e.g., presence of multiple affixes, as in *ruler-ship*), and summarized using common psycholinguistic variables (e.g., length, frequency). This paper also presents an example analysis comparing the lexical decision response times between compound words, pseudo-compound words, and monomorphemic words. Pseudo-compounds and monomorphemic words did not differ in response time, and both groups had slower response times than compound words. This analysis replicates the facilitatory effect of compound constituents during lexical processing, and demonstrates the need to emphasize the pseudo-constituent structure of pseudo-compounds to parse their effects. Further applications of LaDEP include both psycholinguistic studies investigating the nature of human word processing or production and educational or clinical settings evaluating the impact of linguistic features on language learning and impairments. Overall, the items within LaDEP provide a varied and representative sample of the population of English pseudo-compounds which may be used to facilitate further research related to morphological decomposition, lexical access, meaning construction, orthographical influences, and much more.

## Introduction

Large-scale databases of lexical items have been extremely useful in supporting linguistically diverse research and various experimental paradigms. Databases are available in many different languages, including English (e.g., ELP, Balota et al., 2007; SUBTLEX-US, Brysbaert & New, 2009; LaDEC, Gagné et al., 2019; BLP, Keuleers et al., 2012; MorphoLex, Sánchez-Gutiérrez et al., 2018; CompLex, Schmidtke et al., 2021), Dutch (e.g., GECO, Cop et al., 2017; DLP, Keuleers et al., 2010), Chinese (e.g., Chang et al., 2016; CLP, Tse et al., 2017), French (e.g., MEGALEX, Ferrand et al., 2018; LPPC-fMRI, Li et al., 2022; MorphoLex-FR, Malhoit et al., 2020), multiple languages at once (e.g., CELEX by Baayen et al., 1995, which contains English, Dutch, and German; MECO by Siegelman et al., 2022, which contains 13 European languages such as English, Finnish, Greek, Turkish, and Estonian), and many more (see, for example, the Center for Reading Research website, 2023). Databases can serve a range of experimental tasks. Some databases are set up to readily support lexical decision experiments (e.g., Balota et al., 2007; Keuleers et al., 2010, 2012; Malhoit et al., 2020; Sánchez-Gutiérrez et al., 2018; Tse et al., 2017), while others may be applied to eye-tracking (e.g., Cop et al., 2017; Schmidtke et al., 2021; Siegelman et al., 2022), naming (e.g., Balota et al., 2007; Chang et al., 2016), or listening comprehension (e.g., Li et al., 2022), to name a few possibilities.

Regarding the interests of the current project, some databases may be specifically used to support research in English derivational morphology (e.g., Sánchez-Gutiérrez et al., 2018), compound words (e.g., Gagné et al., 2019; Juhasz et al., 2015; Kim et al., 2018; Schmidtke et al., 2021), and word processing broadly (e.g., Baayen et al., 1995; Balota et al., 2007; Brysbaert & New, 2009; Keuleers et al., 2012; Siegelman et al., 2022). Researchers have found them beneficial for evaluating the influence of different morphological constructions and psycholinguistic characteristics on word processing and production, as well as facilitating stimuli selection or pseudo-word creation for experiments. However, no similar pseudo-compound database yet exists that contains a large, representative sample of items or variables useful for morphological processing analyses. The goal of the current project was to construct a database, the Large Database of English Pseudo-compounds (LaDEP), that contains a systematically identified, broad set of several thousand pseudo-compound words. The pseudo-compounds in LaDEP are existing English words that mimic, but do not truly possess, a compound structure (e.g., *carpet* or *bigram*). With this in mind, we consider how this database may inform theoretical concepts such as lexical access, morphological decomposition, and the role of morphological and orthographic representations in lexical processing.

In addition to this comprehensive set of pseudo-compound items, LaDEP includes variables related to the psycholinguistic features of the items (e.g., length and frequency) as well as their morphological and pseudo-morphological features (e.g., presence of affixes, plurality). This database may be applied to multiple experimental paradigms, as it is intended to aid in the selection of stimuli and provide easy access to variables relevant to pseudo-compounds. We begin by providing a brief overview of previous research on pseudo-compounds and then move into a discussion of both the established and novel psycholinguistic variables that are relevant for studying pseudo-compounds. Next, we address how the pseudo-compounds in LaDEP were obtained, and, subsequently, how they were coded and categorized by trained researchers for several features related to their status as a pseudo-compound (e.g., plurality, length, presence of bound affixes). We also present the psycholinguistic properties of these items and an example analysis to demonstrate how users may implement LaDEP to create and design experiments.

Pseudo-morphological structures, such as those seen in pseudo-compound words (e.g., *pantry → pan-try*), pseudo-affixed words (e.g., *corner → corn-er*) and non-words (e.g., *moonhoney*), have been used as an experimental manipulation or a control group to parse the effect of true morphology on complex word recognition and processing (e.g., Leminen et al., 2019). For example, nonwords with a pseudo-compound structure (e.g., *moonhoney*, which is created by switching the morphemes of an existing compound, *honeymoon;* Crepaldi et al., 2013), have been used to examine whether word recognition is sensitive to positional constraints (e.g., Crepaldi et al., 2013). Pseudo-affixation (e.g., *corner*) has been used as a counter-case to true affixation (e.g. *teacher*) to evaluate the presence, timing, and extent of morphological decomposition, and how this interacts with orthographic and semantic information (e.g., Marslen-Wilson et al., 2008; Rastle et al., 2004; Schmidtke et al., 2017; Taft, 1981; Whiting et al., 2013). Similar to pseudo-affixed words, pseudo-compounds have been used as a helpful counter-case to true compounding (e.g., *pantry* vs. *pancake;* Chamberlain et al., 2020; Gagné et al., 2018). Unlike pseudo-affixed words, however, pseudo-compounds contain two pseudo-constituents that are free morphemes with their own extensive morphological, orthographic, and semantic representations (e.g., *pan* and *try* in *pantry*) and thus provide a unique opportunity to evaluate how and when the morphological structure is computed, and how this influences word recognition and processing.

There is not yet a publicly available large database of pseudo-compounds (i.e., existing words, such as *pantry* and *carpet*, that have a pseudo-compound structure; e.g., pan + try or car + pet), and the lack of such a resource makes it difficult to readily incorporate these useful items into research designs. First, using automated code to randomly concatenate words together will yield many non-word pseudo-compounds (e.g., *furcage*) and comparatively fewer real-word pseudo-compounds (e.g., *furrow*). This may be mitigated by cross-referencing with current databases of real words to remove the non-word items; however, true morphological functioning and orthographic coincidence cannot be differentiated using automatic search engines. Similarly, searching for word+word items in existing databases yields both pseudo-compounds and true compounds. Thus, each item must be manually inspected—a difficult and time-consuming task. Alternatively, one might attempt to generate pseudo-compounds based on what comes to mind, but this method is prone to bias (especially recency and availability biases); thus, a set of items obtained in this manner is unlikely to be representative of the population of pseudo-compounds. Therefore, a database of several thousand word-word pseudo-compound items further summarized by psycholinguistic variables is a valuable resource for facilitating further research on compound structure, in particular, and morphologically complex word structure in general.

### Pseudo-compound words in the literature

The term *pseudo-compound* can refer to different types of constructions. Some researchers have defined pseudo-compounds as non-words that are formed by combining two existing words (e.g., *trowbreak* from Taft & Forster, 1976; *houndwork* from MacGregor & Shtyrov, 2013; see also Bronk et al., 2013), an existing word and a non-word (e.g., *sunkib* from Lima & Pollatsek, 1983; see also Taft & Forster, 1976), or two non-words (e.g., Hanssen et al., 2013), or by transposing the constituents of an existing compound (e.g., *moonhoney* from Crepaldi et al., 2013). Other researchers have used existing words, but still there are differences in what is defined as a pseudo-compound. Some make letter transpositions or alterations to existing compounds to create pseudo-compound stimuli (e.g., *cucpake* for *cupcake*; Stites et al., 2016) which turn compounds into non-words. Some have used words with one pseudo-constituent that corresponds to an existing word and one pseudo-constituent that is a non-word (e.g., *trom-bone* in Monsell, 1985), while others have used words where both pseudo-constituents correspond to existing words (e.g., *carpet* in Inhoff, 1989; *patriot* in Gagné et al., 2018; *herring* in Sandra, 1990). In addition to facilitating different research questions and conclusions, the use of non-word pseudo-compounds may be particularly common because real-word pseudo-compounds are difficult to systematically identify.

While any type of pseudo-compound can be useful depending on the particular line of research, LaDEP contains word-word pseudo-compounds because this definition most closely resembles the structure of true compound words. Specifically, these items are real words (and, thus, unlike non-words, have a lexical representation) that orthographically contain two free morphemes with their own set of semantic and psycholinguistic features, but lack the constituent structure characteristic of real compounds (i.e., *pantry* is not composed of the words *pan* and *try,* unlike *snowball* or *strawberry*). For example, *sea* and *son* are morphemes in English, but they do not function as morphemes in the word *season*. Although this type of pseudo-compound has largely been investigated in the visual modality (Christianson et al., 2005; Gagné et al., 2018; Gagné & Spalding, 2016; Inhoff, 1989; Monsell, 1985; Sandra, 1990; Shoolman & Andrews, 2003), some studies in the auditory domain have included pseudo-compounds that are non-words or have non-word constituents (e.g., MacGregor & Shtyrov, 2013). Like non-word pseudo-compounds, the real-word items in LaDEP may be used to support research using a variety of experimental methods and paradigms, such as eye tracking, electroencephalography (EEG), and lexical decision tasks.

Further complicating the varying definition and study of pseudo-compounds is the fact that studies on pseudo-compound words (of any definition) are uncommon. These constructions may also be used as control items rather than the primary manipulation in experiments. For example, Bronk et al. (2013) used German pseudo-compounds (described as “compound non-words”) as an experimental control so that the authors could make conclusions about the nature of decomposition (automatic or not) and the subsequent influence of true morphology and semantic transparency. Some pseudo-compounds were the non-word combination of two real words (e.g., *Pianotasse, *pianocup*), and others were misspelled compound words (e.g.,*Blamentepf, *flewerpat* for *flowerpot*). This study found that compounds had a processing advantage over pseudo-compounds and monomorphemic words for transparent compounds only. Further, the inclusion of pseudowords which contained two lexical items, such as “*Pianotasse,” did not remove the processing advantage for semantically transparent compound words, but did remove it for semantically opaque compounds. As another example, Monsell (1985) used pseudo-compounds as a control to determine whether, and which, constituent effects were lexical and which were orthographic or phonological in nature. In this study, pseudo-compounds were real words with either two real-word constituents (e.g., *furlong*) or one real-word and one non-word constituent (e.g., *trombone*). He found that, when primed with the constituents, people were slower to respond to pseudo-compounds than to compounds. This result suggested that the effects seen in compound words were lexical rather than solely orthographic or phonological.

The unique mimicry of a compound morphological structure makes word-word pseudo-compounds useful for developing theories of morphological processing. For example, if the pseudo-constituent representations become available during processing, this could delay the linguistic system and require that these erroneous representations are suppressed. On the other hand, other theories might predict that the system accesses the whole word first, and thus the pseudo-morphemes are never accessed and do not require any additional processing steps. Either case extends previous research regarding the way in which words are processed and the order in which different types of information become available (e.g., Creemers et al., 2020; Crepaldi et al., 2013; Gagné et al., 2018; Manelis & Tharp, 1977; Rastle et al., 2004; Sandra, 1990; Shoolman & Andrews, 2003).

Increasingly, researchers are using pseudo-compounds as experimental targets rather than solely control words. Some early research used compound non-words and pseudo-affixed words as primary experimental manipulations (Lima & Pollatsek, 1983; Taft & Forster, 1976). Other research has directly compared the processing of compounds and pseudo-compounds (e.g., Bronk et al., 2013; Crepaldi et al., 2013; Sandra, 1990; Shillcock, 1990; Shoolman & Andrews, 2003). These studies demonstrate how pseudo-compounds can provide a valuable test case for psycholinguistic research. For example, researchers investigating the effect of decomposition within the processing of multimorphemic words found that extraction of embedded morphemes occurred for compounds as expected, and also for pseudo-compounds (Chamberlain et al., 2020). The orthographic units, or pseudo-morphemes, that were recovered within pseudo-compounds were unhelpful and hindered their processing, while the recovery of the constituents aided the processing of compound words and multimorphemic words where the units were truly productive (Chamberlain et al., 2020). Similarly, another study showed that when the target (e.g., *cash*) was not a truly productive morpheme in the prime (e.g., *cash* is not a productive morpheme in the pseudo-compound *cashmere*), it became more difficult for participants to identify the target *cash* as a word after being presented with the pseudo-compound (Gagné et al., 2018). On the other hand, when the target (i.e., first constituent) was a true morpheme (e.g., *cash* is a productive morpheme in the compound *cashcard*), it was much easier for participants to identify the target *cash* as a word after being presented with the compound *cashcard* compared to an unrelated word (Gagné et al., 2018). When the experimental behaviour of pseudo-compounds differs from compounds, multimorphemic words, and monomorphemic words, as in these studies, this suggests that there are aspects of morphological processing which occur automatically and must be adjusted when the constituent information is erroneous. This pattern provides an opportunity for researchers to better understand what happens when compound words, and other types of multimorphemic words, are processed.

Overall, it is uncommon for pseudo-compounds to be used as more than an experimental control, and studies might not be directly comparable due to differing stimulus sets and definitions of what constitutes a pseudo-compound (e.g., nonexistent words such as *dustworth* or *trowbreak* in Taft & Forster, 1976, vs. an existing word such as *carpet* in Gagné et al., 2018). Having a representative set of items of word-word pseudo-compounds that are themselves existing English words will facilitate the unbiased selection of items and research with this type of pseudo-compound, which can inform theories related to morphological processing, conceptual combination, orthographic influences on word processing and production, and much more.

### Psycholinguistic features of pseudo-compounds

Previous research has shown that length (i.e., number of letters), frequency, and positional family size (analogous to morphological family size) all influence the processing of pseudo-compounds and compounds. Thus, these variables were selected for inclusion in this project. Constituent and full-word lengths have been shown to influence language comprehension and word memory such that longer lengths are associated with longer processing times (e.g., Barton et al., 2014; Bertram & Hyönä, 2003). Frequency effects are well established, especially in fields related to language comprehension and language acquisition, such that higher-frequency words are more readily acquired and are associated with shorter response times (e.g., Hyönä & Olson, 1995; Monsell, 1985); this pattern is similarly seen in more frequent compounds and constituents (e.g., Juhasz, 2006; Marelli & Luzzatti, 2012; Schreuder & Baayen, 1997). Positional family size refers to the number of words which share a pseudo-morpheme in a particular position. For example, with respect to the pseudo-morpheme ANT, the first constituent family size would count all the ANT+X words (e.g., *antelope*), and the second constituent family size would count all the X+ANT words (e.g., *fondant*). Morphological family size, which is the same concept as positional family size but referring to true morphemes, has been shown to be an important explanatory variable in the study of multimorphemic words (e.g., Baayen et al., 1997b; De Jong IV et al., 2000; Feldman & Pastizzo, 2003; Nikolaev et al., 2019; Schreuder & Baayen, 1997).

In conjunction with identifying the frequency, length, and positional family size of the items in LaDEP, we also manually coded the items to allow users to identify additional morphological and pseudo-morphological characteristics of the pseudo-compound. Specifically, pseudo-compounds may contain affixes that double as free morphemes and are either functioning (e.g., -*age* in *linkage*) or not functioning (e.g., -*age* in *damage*). Just as pseudo-compounds yield different effects from compound words (e.g., Gagné et al., 2018; Taft & Forster, 1976), pseudo-affixed words show different effects during processing when compared to truly affixed words (e.g., Rastle et al., 2004). Consideration of these functioning and non-functioning affixes renders three types of pseudo-compounds: (1) pseudo-compounds where at least one pseudo-constituent is a functioning affix, (2) pseudo-compounds where at least one pseudo-constituent is a non-functioning affix, and (3) pure pseudo-compounds where neither pseudo-constituent could be an affix. Current research investigating the effect of these pseudo-compound and affixed representations has suggested that there are differences between these three types of pseudo-compounds in both comprehension and production (Auch et al., 2023).

Similarly, a pseudo-compound may contain *combining forms.* Combining forms are similar to derivational affixes in that they combine with word stems but differ from derivational affixes in that they alter the meaning of the word rather than its word class (Lehrer, 1998; e.g., *techno-* in *technobabble*). The variables representing these concepts will be further discussed in the Method section. These variables have been coded and included because there is some evidence in the literature to suggest that these distinctions might be relevant to processing (Fradin, 2000; Iacobini, 1997; Lehrer, 1998). Like affixes, combining forms may be used in combination with stems, and some share orthography and etymology with free morphemes. Unlike affixes, the meaning of a combining form may be similar to its free morpheme (e.g., *radio-* means related to radiation or rays, and a *radio* is an object which functions using radiofrequency radiation) or quite different from its unbound counterpart (e.g., *pan*- denotes “all” or “everything,” while a *pan* is an object used for cooking). Combining forms are not commonly evaluated from a psycholinguistic standpoint, but they are constructions distinct from free morphemes or affixes (Iacobini, 1997; Lehrer, 1998).

The word-word pseudo-compounds in LaDEP are existing English words and, thus, possess several psycholinguistic features which could impact human processing and production. Ultimately, the inclusion of these variables will aid researchers hoping to answer specific theoretical questions. As with other lexical constructions, these psycholinguistic features may be manipulated to evaluate various predictions and theoretical frameworks.

## Method

### Creation of a set of English pseudo-compounds

The Large Database of English Pseudo-compounds (LaDEP) contains words that have two free morphemes, but are not actually compounds (e.g., *carpet* or *lotion*); they do not have a compound morphological structure. For example, even though the word *bigram* contains the English morphemes [big] and [ram], its morphological structure is [[bi]+[gram]] rather than a compound structure ([big]+[ram]). The following sections will give a general overview of how the items in LaDEP were obtained and retained. In brief, our collection of pseudo-compound words was obtained by concatenating potential constituents into word-word items and identifying which items were real English words, but not compounds.

### Creating word-word items

The creation of the word-word items was a two-step process and was completed simultaneously with the creation of the Large Database of English Compounds (LaDEC; Gagné et al., 2019). First, potential constituents were gathered from the items in the British Lexicon Project (Keuleers et al., 2012), the set of all nouns and adjectives in the English Lexicon project (Balota et al., 2007), and Mathematica’s Word Dictionary and WordData set (Wolfram Research Inc., 2019). The length of the constituents was restricted to 3–10 letters, and words with both an affix and a noun sense (e.g., *hood* is both a suffix and a noun) were included. The resulting 76,424 constituents were then concatenated into a list of all possible word-word combinations, resulting in more than 5.8 billion items. All non-words and non-nouns in this set of word-word items were removed by only extracting those which appeared in the set of nouns in WordNet, the Mathematica dictionary of English words, the English Lexicon Project (Balota et al., 2007), or the British Lexicon Project (Keuleers et al., 2012). This resulted in 28,630 items, and this set was further restricted to 16,697 items by only maintaining items classified as nouns in WordNet. We chose to focus on nouns to ensure that the set of items comprising LaDEP could be easily compared to one another and to other databases, and simultaneously be manageably hand-coded by members of the research team. This set of 16,197 items included both true compounds and pseudo-compounds.

### Identifying non-compound items as pseudo-compounds

The final stage of obtaining the set of items involved the manual coding of the 16,697 word-word items by trained research assistants as to whether each item was a true compound or not. The 8956 compound items were included in the Large Database of English Compounds (Gagné et al., 2019). The 7741 non-compound items were excluded from the LaDEC project and instead formed the basis for the current project.

The specification and further coding of LaDEP began after the completion and publication of the LaDEC database. During the creation of LaDEP, the non-compound items were again screened for the presence of compounds while simultaneously being coded for features relevant to pseudo-compounds. An additional 286 items were identified as compound words after consulting the Oxford English Dictionary (OED; Oxford University Press, 2021). One item, *cranberry*, was kept in LaDEP, as its status as either a compound or a monomorphemic word has long been debated in the linguistic literature (e.g., Bolinger, 1948; Carstairs-McCarthy, 2017). Some items, upon consulting the OED or other online dictionaries (e.g., Merriam-Webster), were noted to be spelled incorrectly (e.g., *adesite* instead of *andesite*), were an alternative spelling (e.g., *milage* and *mileage*), or were a derivation not listed in a dictionary but feasibly understood by English speakers (e.g., *appetizingness*). These items were kept in LaDEP as they were a product of how the items were obtained and are present in other databases (e.g., Mathematica’s Word Dictionary). This procedure resulted in the set of 7455 pseudo-compound items which became the current database, the Large Database of English Pseudo-compounds (LaDEP). The true morphological structure of the pseudo-compound items in LaDEP may be either monomorphemic (e.g., *pantry*) or multimorphemic (*e.g., ejection*). Similarly, the pseudo-constituents may be mono- or multimorphemic (e.g., *tar* in *target* vs. *ruler* in *rulership*). The presence of these morphological structures and other relevant morphological features were coded in LaDEP.

### Coding features of pseudo-compounds

Each pseudo-compound item was coded for multiple features related to its status as both a pseudo-compound and a monomorphemic or multimorphemic word. Two research assistants conducted the coding over the course of 2 years, and all items were coded by consulting the Oxford English Dictionary (OED; Oxford University Press, 2021) as the primary source. Coding generally involved searching for the definition and origin of the whole item, the first pseudo-constituent, and the second pseudo-constituent and evaluating each for the specific feature described. The majority of features coded were based on objective information (e.g., presence of an affix entry for the word). Secondarily, if the OED did not contain sufficient information, coders first consulted the Online Etymology Dictionary (Etymonline, 2021) and then Wiktionary.com. In the case that the sources disagreed, the OED information was used. If there was ambiguity in the information presented in the OED, the item(s) were discussed between the two research assistants and the broader research team until consensus was reached. Items present within the OED are marked by the variable *inOED.*

#### Orthographic presence of bound affixes and combining forms

The initial level of coding denoted whether the first pseudo-constituent or the second pseudo-constituent doubled orthographically as an affix or combining form. The variable names for affixes and combining forms, respectively, were *bound_location* and *combine_form.* These variables did not mark affixes or combining forms in words that were not parsed at the pseudo-constituent boundary (e.g., the affixes “-ship” and “re-” were not marked in *scholars+hip* or *real+location*). Additionally, they did not mark affixes or combining forms contained within the pseudo-constituent (e.g., *-er* in *rulership*). Both *bound_location* and *combine_form* marked which pseudo-constituent was functioning as the bound element (i.e., pseudo-C1 or pseudo-C2 or both). These variables also considered position; that is, because *-let* is a suffix, this constituent was only marked as an affix in *bound_location* if the second pseudo-constituent was *let* (see Crepaldi et al., 2010, 2013, for examples of evidence for positional effects of stems and affixes, even for pseudo-words). If the first pseudo-constituent was *let,* this variable was not marked. Neither variable distinguished whether the affix or combining form was morphologically present within the pseudo-compound (e.g., *booklet*), or whether it was an orthographic coincidence (e.g., *scarlet*).

#### Morphological functionality of bound affixes and combining forms

The previous section discussed variables which marked the orthography present within the pseudo-compound. This section presents two corresponding variables which marked whether an orthographic affix (*derived_affix*) or combining form (*fxn_cf*) was morphologically present within the pseudo-compound. That is, these variables marked orthographic coincidences as null and ignored items which were not marked as having an orthographic affix or combining form (i.e., these were missing values in the variable). These codings differentiated between *linkage [[link]+[-age]]* and *damage,* or *pantheist [[pan-]+[theist]]* and *pantry*. Like the previous variables, this coding was positionally bound. That is, a prefix or initial combining form which coincided with the first pseudo-constituent was marked. If the etymology indicated that an affix or combining form combined with a stem to form the pseudo-compound (e.g., [[link]+[age]]), the variables in LaDEP were marked as affirmative and for the specific position of the affix or combining form.

#### Borrowed affixes

This variable (*borrowed_affix*) denoted whether an affix identified by the variable *bound_location* was borrowed from another language. That is, a borrowed affix was one that was present in a previous language, such as French or Latin, attached to the original stem in that language, and similarly transferred to English as an affix (e.g., *-ion* in *ejection*). This borrowing was determined by the word origin as described in the OED. Borrowed affixes in English may be active, where the affix can be separated to form the corresponding stem (e.g., -*ion* in *ejection* is borrowed but can be removed to form the stem *eject*), or inactive, where this separation does not form the corresponding stem in English (e.g., *-ion* in *accordion* is borrowed and can be stripped to form *accord*, but this is not the English stem of *accordion*).

#### Altered stems

When an affix combines with a stem, the form of the stem may change due to phonotactic or orthographic influences of the language. For example, [dose]+[age] forms *dosage* rather than “*doseage”*; thus, the form of the stem has been altered. This may be an addition to, subtraction from, or change in the stem’s form—that is, its letters or sounds. The LaDEP variable *stem_alt* marked these alterations as either occurring or not for each item that contained a functioning affix (e.g., *wastage* vs. *wreckage*), as denoted by the variable *derived_affix*. Items marked for *stem_alt* may also contain borrowed affixes, provided that the affix is still active (e.g., *erosion*). This variable was not marked if the supposed affix was not functioning (e.g., *million*) or was an inactive borrowing (e.g., *provision*).

#### Multiple affixes and multiple combining forms

The pseudo-compounds in LaDEP were parsed into two pseudo-constituents that may be multi- or monomorphemic words. While the previous morphological variables in LaDEP account for the morphological structure of items that were parsed at their morphemic boundary (e.g., *select+ion*), they fail to account for items which contain affixes or combining forms within their pseudo-constituents (e.g., *react+ant* can be further parsed into *re- act -ant; auto+radiograph* can be parsed into *auto- radio- -graph*). Items in LaDEP with more than one derivational affix were marked affirmatively in *multiple affixes*, while items with more than one combining form were marked affirmatively in *multiple_cf*. Inflectional affixes, such as *-s, -ing,* or *-ed*, were ignored. For example, the item *youthful+ness* was marked as having multiple derivational affixes (i.e., *-ful* and *-ness*), while *eye-let* only has one, and *car-pets* has none.

#### Plurality

Many of the items in LaDEP were plural. As researchers may wish to distinguish between singular and plural items, we included a categorization that denoted whether the item is plural. In some cases the pseudo-compounds were listed with a non-plural counterpart (e.g., *tenant* and *tenants*). In other cases, only the plural form of the word was a pseudo-compound because the plurality resulted in an orthographic alteration that corresponded to an English free morpheme (e.g., *quarter lies* or *come dies*). Irregular plurals (e.g., *hypotheses*) were counted as plural in this variable

### Inclusion of psycholinguistic and linguistic variables

Because the primary goal of LaDEP is to provide a resource to facilitate research on processing and production of pseudo-compound words, we also included variables that represented psycholinguistic and linguistic features previously used by researchers for stimuli selection, analyses, and experimental design.

#### Length and frequency

The length, in number of letters, of the pseudo-compound, first pseudo-constituent, and second pseudo-constituent were calculated for all items in LaDEP. The log_10_ word frequency for the pseudo-compound and the first and second pseudo-constituents were obtained from SUBTLEX-US (Brysbaert & New, 2009) for the items that occurred in both databases. The variables *stim_hasFREQ*, *c1_hasFREQ*, and *c2_hasFREQ* indicate which items were found in the SUBTLEX-US database to allow readers to access this frequency information directly from the database of origin.

#### Positional family size

We calculated the positional family size of the pseudo-constituents in terms of all items included in LaDEP. Here, positional family size refers to the number of items that share the same pseudo-constituent in the same position within the LaDEP database. For example, the positional family size for *ion* in the pseudo-C2 position would equal the number of items in LaDEP that have *ion* as their pseudo-C2.

#### Response time data

We coded whether response time data from the English Lexicon Project (ELP; Balota et al., 2007) and British Lexicon Project (BLP; Keuleers et al., 2012) databases were available for each item. The variables inELP and inBLP indicate which items in LaDEP were found in those respective databases.

## Results

### Descriptive statistics

**Fig. 1:**
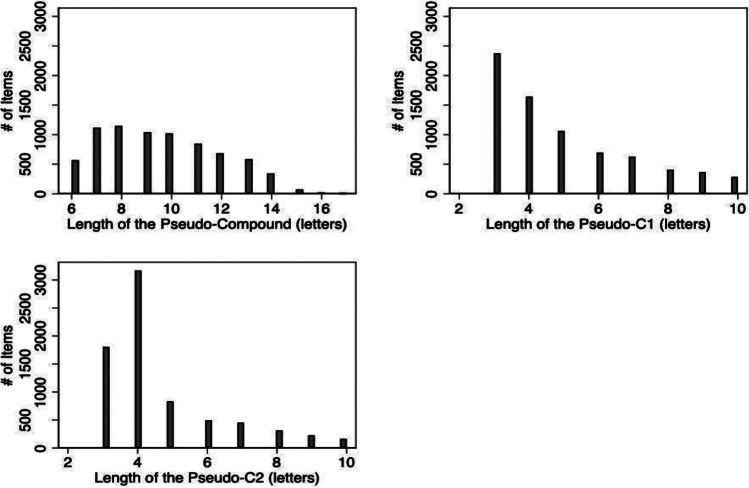
Length (number of letters) of the pseudo-compound, first pseudo-constituent, and second pseudo-constituent for all items in LaDEP

**Fig. 2:**
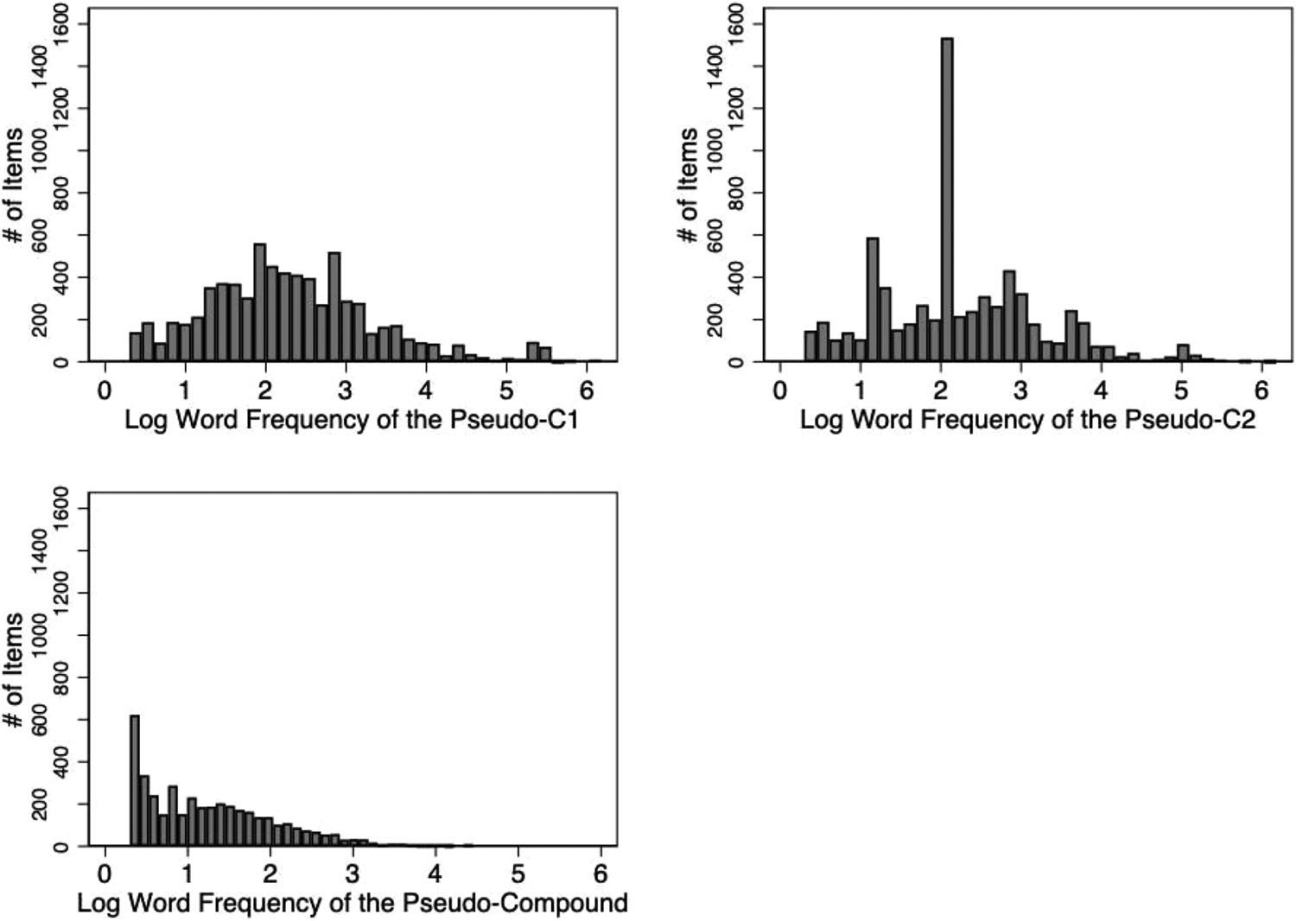
Log word frequency values taken from SUBTLEX-US (Brysbaert & New, 2009) for the first pseudo-constituent, second pseudo-constituent, and the full pseudo-compound

**Fig. 3:**
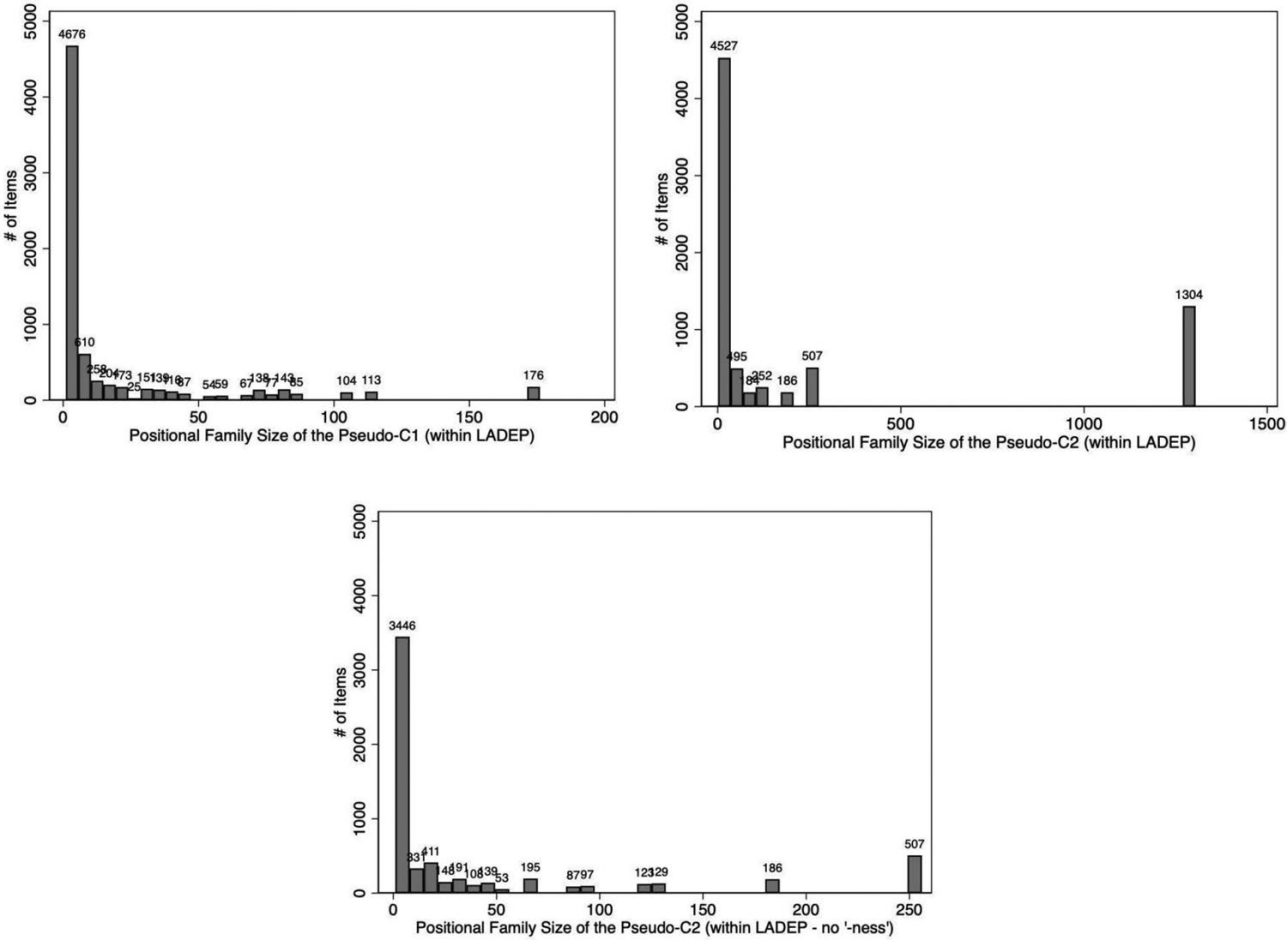
Positional family size of the first and second pseudo-constituents based on items in LaDEP*.* In the third panel, the outlier suffix “ness” has been removed

**Fig. 4:**
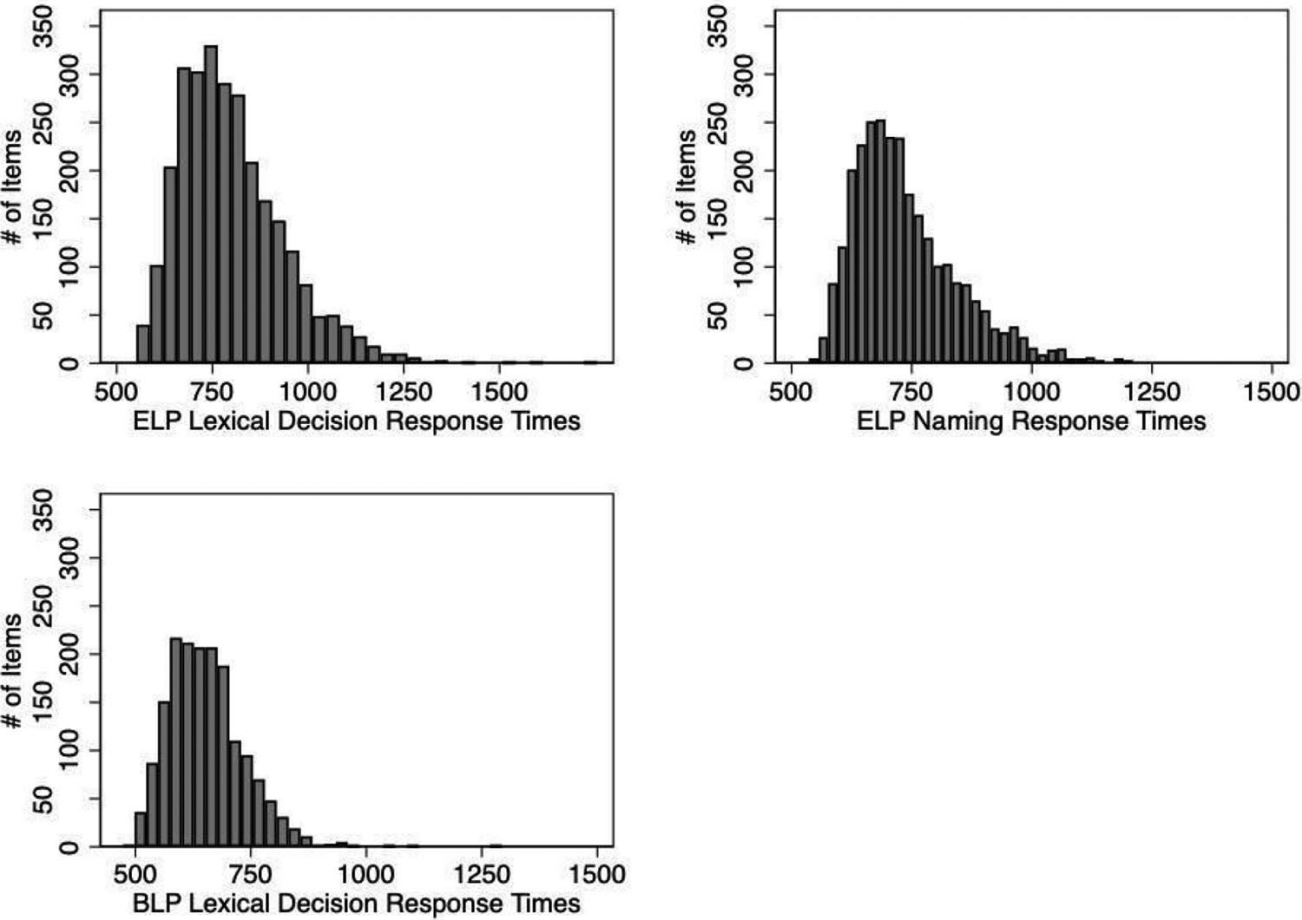
Lexical decision response times (n = 2800) and naming response times (n = 2801) from the English Lexicon Project (ELP; Balota et al., 2007) and lexical decision response times from the British Lexicon Project (n = 1705; BLP; Keuleers et al., 2012) for the pseudo-compound items in LaDEP

Table 1 shows the descriptive statistics for the length, frequency, positional family sizes, and response times of the pseudo-compounds in LaDEP and, where relevant, their pseudo-constituents. The distributions of these variables are represented in Figs. 1, 2, 3 and 4. In creating LaDEP, the length of the pseudo-constituents was constrained to a minimum of 3 characters and a maximum of 10 characters. Thus, the pseudo-compounds could range from 6 to 20 characters in length; the resultant minimum length of the pseudo-compounds was 6 and the maximum length of the pseudo-compounds was 17. On average, the length of the first and second pseudo-constituents was 5.0 letters (SD = 2.0) and 4.6 letters (SD = 1.7), respectively. The mean length of the overall pseudo-compound was 9.6 letters (SD = 2.3). Figure 1 shows the distribution of lengths for the first pseudo-constituent (pseudo-C1), the second pseudo-constituent (pseudo-C2), and the pseudo-compound.

**Table 1:** Summary statistics for length, frequency, positional family size, and response time during lexical decision and naming. Frequency values were obtained from the SUBTLEX-US database (Brysbaert & New, 2009), and response times were obtained from the English Lexicon Project (ELP; Balota et al., 2007) and British Lexicon Project (BLP; Keuleers et al., 2012)

Variable	*n*	*M*	*SD*	min	max
Pseudo-C1 length (letters)	7455	5.0	2.0	3	10
Pseudo-C2 length (letters)	7455	4.6	1.7	3	10
Pseudo-compound length (letters)	7455	9.6	2.3	6	17
Pseudo-C1 log_10_ word frequency (SUBTLEX-US)	7147	2.3	1.1	0.3	6.2
Pseudo-C2 log_10_ word frequency (SUBTLEX-US)	6970	2.3	1.0	0.3	6.2
Pseudo-compound log_10_ word frequency (SUBTLEX-US)	4121	1.3	0.8	0.3	4.5
Pseudo-C1 positional family size	7455	19.2	35.9	1	176
Pseudo-C2 positional family size	7455	263.7	483.9	1	1304
ELP lexical decision response time (ms)	2800	801	139	552	1756
ELP naming response time (ms)	2801	738	109	536	1211
BLP lexical decision response time (ms)	1705	654	82	473	1293

When present, log_10_ frequency data from SUBTLEX-US (Brysbaert & New, 2009) were gathered for the pseudo-C1 (*n* = 7147; M = 2.3, SD = 1.1), the pseudo-C2 (*n* = 6970; M = 2.3, SD = 1.0), and the pseudo-compound (*n* = 4121; M = 1.3, SD = 0.8). Figure 2 illustrates the distribution of the SUBTLEX-US log_10_ frequency values for the pseudo-C1, pseudo-C2, and pseudo-compound.

We calculated positional family sizes relative to the other items in LaDEP for all pseudo-constituents. On average, the first pseudo-constituents had approximately 19 items that shared that same first pseudo-constituent (M = 19.2, SD = 35.9). The second pseudo-constituent had a higher-centered and wider distribution of positional family sizes (M = 263.7, SD = 483.9). The upper panels of Fig. 3 show the distribution of positional family sizes for both constituents. From this figure, it is clear that the values for the second pseudo-C2 represent the influence of an outlier item, the pseudo-C2 *ness*, which has a positional family size of 1304 items. The lower panel of Fig. 3 shows the distribution of positional family sizes for the pseudo-C2 after the outlier *ness* was removed. After removing this item from the analysis, the remaining 6151 items had an average positional family size of 43.2 (SD = 75.5). That is, on average, the pseudo-compounds shared the same second pseudo-constituent with 43 total items.

A total of 2801 of the items in LaDEP were included in the English Lexicon Project (ELP; Balota et al., 2007). We obtained the ELP lexical decision response times, in milliseconds, for 2800 of these items (M = 801, SD = 139). ELP naming response times, in milliseconds, were obtained for all 2801 items (M = 738, SD = 109). A total of 1721 of the items in LaDEP were present in the British Lexicon Project (BLP; Keuleers et al., 2012). We obtained BLP lexical decision response times, in milliseconds, for 1705 of these items (M = 654, SD = 82). Figure 4 shows the histograms of these response time variables from the ELP and BLP for the pseudo-compounds in LaDEP.

The distribution of response times (from Keuleers et al., 2012; Balota et al., 2007) and frequency (from Brysbaert & New, 2009) for the items in LaDEP were similar to the reported distributions of these variables in their database of origin. The mean ELP naming and lexical decision response times for the subset of items in LaDEP were within 20 milliseconds of those reported in Balota et al. (2007). The distribution of LaDEP items for the BLP responses times was similarly centered (both M = 654 ms) but had a smaller range than those reported by Brysbaert and New (2009; 473–1293 ms vs. 300–1617 ms). The distribution for the log_10_ word frequency pseudo-compounds was also similar (within 0.06 for both mean and standard deviation) to the log_10_ word frequency distribution of the complete set of items in SUBTLEX-US (M = 1.19, SD = 0.84, from Brysbaert & New, 2009).

### Features related to affixes and combining forms

The majority of pseudo-compounds in LaDEP contained either a possible bound affix (*n* = 4312), a possible combining form (*n = *919), or both (*n = *141). Table 2 shows the location of these possible bound morphemes and summarizes *derived_affix* and *fxn_cf* which denote whether these possible affixes are truly functioning within the word. From this table, we see that the majority of items with functional affixes had suffixes, while most items with functional combining forms had initial combining forms. Regarding the other manually coded variables, most items in LaDEP were not plural (*n = *5664) and did not contain multiple functional affixes (*n = *6079) or combining forms (*n = *7173). For items that did contain a true affix or combining form, only a small minority underwent alteration of their stems due to this combination (*n = *168). Finally, most of the potential affixes identified in LaDEP were not borrowed from another language (*n = *3166), but any borrowed affixes were more likely to be an active borrowing (*n = *871) than an inactive borrowing (*n = *416).

**Table 2:** Summary table of the manually coded variables *derived_affix* and *fxn_cf*. Variable names are in brackets

	Location of possible bound morpheme (*bound_location OR combine_form*)			
	Pseudo-C1	Pseudo-C2	Both	Total
Location of functional affix (*derived_affix*)				
Neither	316	479	16	811
Prefix	951	–	22	973
Suffix	–	2660	9	2669
Both	–	–	0	0
Location of functional combining form (*fxn_cf)*				
Neither	194	65	5	264
Pseudo-C1	498	–	29	527
Pseudo-C2	–	115	4	119
Both	–	–	150	150

Possible bound morphemes are marked in *bound_location* for affixes and *combine_form* for combining forms

Many of the pseudo-compounds in LaDEP are present in other relevant databases. To aid stimuli selection from LaDEP, specific variables in LaDEP denoted the presence of the pseudo-compound item in the Oxford English Dictionary (*n = *7190; *inOED*; Oxford University Press, 2021), English Lexicon Project (*n = *2801; *inELP*; Balota et al., 2007), British Lexicon Project (*n = *1721; *inBLP*; Keuleers et al., 2012), and SUBTLEX-US (*n = *4121; *stim_hasFREQ*; Brybaert & New, 2009). Users of LaDEP can use these variables to select their items based on their availability in these databases, if desired. LaDEP contains a variety of items with varying lengths, frequencies, positional family sizes, and constituent characteristics that can be used to explore a variety of research questions.

### Example analysis

#### Materials and design

To demonstrate how LaDEP and pseudo-compounds can be incorporated into experimental designs, we completed a simulated experiment using lexical decision response times from the English Lexicon Project (Balota et al., 2007). A total of 2800 items in LaDEP possessed ELP lexical decision times. The experiment utilized three groups: monomorphemic words (e.g., *demise* from ELP), pseudo-compound words (e.g., *pantry* from the current database), and compound words (e.g., *seaman*; from Gagné et al., 2019). We matched items from each word type on length and log_10_ word frequency from SUBTLEX-US (Brysbaert & New, 2009). To limit the scope of the analysis and provide a concise demonstration of the use of LaDEP, only pseudo-compounds which did not possess a derivational affix (e.g., *car-pet*, rather than *link-age*) were included (Auch et al., 2023, expands on the impact of potential and true derivational affixes in pseudo-compounds). Items with plural inflection were included and matched together and similarly matched for length and frequency. These parameters resulted in 462 sets of matches across the three word types; thus, 1386 items were included in the analysis; 120 of these matched sets, or 360 items total, possessed plural inflection.

#### Example analysis results

Data were analyzed using multiple linear regression with ELP lexical decision times as the response variable and the word type as the primary predictor. Length and frequency were included as covariates. The analysis was conducted in Stata 16 (StataCorp, 2019). Table 3 shows the descriptive statistics for the items presented within the current analysis. We fit two models, one where both plural and non-plural matches were allowed and another where the plural matches were removed. Table 4 shows the regression results for all items (Model 1) and those without plural inflection (Model 2).

**Table 3:** Summary statistics for length, frequency, and ELP lexical decision response times for the experimental items. Statistics are additionally split by word type

Variable	*n*	*M*	*SD*	min	max
*All items*	1386				
Length (letters)		7.63	1.20	6	12
log_10_ word frequency		1.62	0.68	.301	3.96
ELP lexical decision time (ms)		762	105	551	1281
*All non-plural items*	1026				
Length (letters)		7.42	1.13	6	12
log_10_ word frequency		1.72	0.69	.301	3.96
ELP lexical decision time (ms)		762	108	551	1281
*Monomorphemic words*	462				
Length (letters)		7.63	1.20	6	12
log_10_ word frequency		1.62	0.69	0.301	3.96
ELP lexical decision time (ms)		771	102	573	1280
*Pseudo-compound words*	462				
Length (Letters)		7.63	1.20	6	12
log_10_ word frequency		1.62	0.68	0.301	3.79
ELP lexical decision time (ms)		774	113	562	1281
*Compound words*	462				
Length (Letters)		7.63	1.20	6	12
log_10_ word frequency		1.62	0.69	0.301	3.77
ELP lexical decision time (ms)		742	98	551	1162

**Table 4:** Standardized regression coefficients with standard errors (in parentheses) using word type to predict lexical decision times from the English Lexicon Project (Balota et al., 2007)

	Model 1	Model 2
	All items	Non-plural
Wordtype		
Pseudo-compound	3.30	10.29
	(5.79)	(6.73)
Compound	−28.82^***^	−21.39^**^
	(5.79)	(6.73)
Length	12.59^***^	17.39^***^
	(1.97)	(2.44)
log_10_ word frequency	−79.52^***^	−88.23^***^
	(3.46)	(4.01)
_cons	803.61^***^	788.14^***^
	(16.53)	(19.29)
*N*	1386	1026
Adj *R*^2^	0.304	.340
Partial ε^2^		
Wordtype	0.0249^a^	0.0202^a^
Length	0.0281^a^	0.0466^a^
log_10_ word frequency	0.276^b^	0.321^b^

^*^p < 0.05, ^**^p < 0.01, ^***^p < 0.001

^a^Small effect based on Cohen (1992) criteria

^b^Large effect based on Cohen (1992) criteria

Monomorphemic words function as the base level of word type. Length and frequency are included as control variables

The overall regression model was significant in both Model 1 (*R*^2^_adj_ = 0.304, *F*(4, 1381) = 151.99, *p* < 0.001) and Model 2 (*R*^2^_adj_ = 0.340, *F*(4, 1021) = 133.13, *p* < 0.001). Moreover, the pattern of significant predictors was identical for both models; thus, we choose to focus on Model 1 and expound on those results here. Between the different word types, only compound words significantly predicted response time (β = −28.82, *p* < 0.001). That is, compound words were, on average, responded to approximately 29 milliseconds faster than both pseudo-compounds and monomorphemic words. This result aligns with previous research suggesting that the constituent structure of compound words facilitates their lexical access, thus facilitating the lexical decision response time of participants (e.g., Christianson et al., 2005; Duñabeitia et al., 2009; Fiorentino & Fund-Reznicek, 2009; Gagné et al., 2018; Shoolman & Andrews, 2003).

In the current analysis, pseudo-compounds were not a significant predictor of ELP lexical decision response time relative to monomorphemic words. Without any additional manipulations, it’s difficult to determine the specific internal process behind this result. For example, it is possible that the pseudo-constituents are not accessed and the monomorphemic whole-word structure of the pseudo-compounds is accessed directly. On the other hand, the pseudo-constituents may indeed be retrieved but are rapidly suppressed due to psycholinguistic factors (e.g., familiarity), such that there is little detriment to overall processing speed. Previous research has shown support for both the former (e.g., Sandra, 1990; Shoolman & Andrews, 2003) and the latter (e.g., Gagné et al., 2018). Neither case can be differentiated from the other, or any other theoretical possibility, based on simple lexical decision alone. Many studies with pseudo-morphemic structures employ masked priming (e.g., Duñabeitia et al., 2009; Rastle et al., 2004) to emphasize these erroneous structures and evaluate how the psycholinguistic system handles this information. Nonetheless, this analysis with simple lexical decision data demonstrated how LaDEP may be used to facilitate stimuli selection and experimental design.

## General discussion

The Large Database of English Pseudo-compounds (LaDEP) contains nearly 7500 pseudo-compound items that researchers may use to build experiments, select stimuli or control items, answer theoretical questions, and support their research programs. LaDEP is a useful resource for researchers investigating the influence of orthographic, morphological, and compositional information on word processing and production. While pseudo-compounds are certainly useful as experimental controls, they also can provide information about the linguistic organization and access through their own experimental manipulation (e.g., Crepaldi et al., 2013; Gagné et al., 2018; Lima & Pollatsek, 1983; Shoolman & Andrews, 2003). The items and variables presented in LaDEP may help generate ideas and stimuli for various experimental manipulations for evaluating theories, such as those related to morphological decomposition.

One particularly useful attribute of LaDEP is its representation of the population of English word-word pseudo-compounds. LaDEP possesses a large set of items, which makes it more likely to be representative of the entire population of this word type in English. Our results show a strong similarity in the distributions of frequency values and response times between the items presented in LaDEP and the original databases from which these values are derived (ELP, BLP, & SUBTLEX-US). These similarities suggest that the items presented here are representative samples of the original set of items in these databases. Additionally, the variables of length and positional family size have been calculated based on the items contained in LaDEP and thus are similarly likely to be representative of the population of word-word pseudo-compounds that are existing English words. Ultimately, LaDEP provides an opportunity to select their stimuli from a representative sample of English word-word pseudo-compounds which are themselves existing words.

To facilitate research using this set of items, LaDEP contains variables relevant to pseudo-compounds but not thoroughly studied, in addition to well-established variables (e.g., length, frequency). This novel set of variables identifies the characteristics of their constituents and the pseudo-compound as a whole; specifically, the orthographic and morphological presence or absence of affixes and combining forms. These variables together provide a detailed picture of each item in LaDEP and will allow researchers to test novel hypotheses related to the morphological and/or pseudo-morphological features of pseudo-compounds. Users of LaDEP can access variables of length for the pseudo-compound and its constituents (*N* = 7455), frequency (SUBTLEX-US; Brysbaert & New, 2009) for the first pseudo-constituent (*n* = 7147), second pseudo-constituent (*n* = 6970), and pseudo-compound (*n* = 4121), positional family size based on the total number of items in LaDEP (*N* = 7455), and lexical decision (*n* = 2800) and naming (*n* = 2801) response time from the ELP (Balota et al., 2007) and lexical decision response time from the BLP (*n* = 1705; Keuleers et al., 2012).

The current study additionally demonstrated a diversity among pseudo-compounds such that some only map onto a compound structure (e.g., [[car]+[pet]]) whereas others can map onto either a compound structure or an affixed word structure (e.g., [[lot]+[ion]], [[link]+[age]]). Thus, while LaDEP provides an ample set of pseudo-compounds to assess the effects of pseudo-morphological information, it additionally provides more fine-grained information regarding the different potential representations of their pseudo-constituents. This level of information makes the database useful for researchers interested in affixed words. Nearly 4500 items in LaDEP have at least one pseudo-constituent that could be either a free morpheme or an affix (e.g., *-age*, *super-*, *-ion*). Some of these items are truly derived words (e.g., *linkage*) while others are not (e.g., *damage*). This renders different possible combinations of morphological representations: (1) those with a single pseudo-compound representation, such as [[car]+[pet]], (2) those with one derived word representation and one pseudo-compound representation, such as [[link]+[age]], and (3) those with one pseudo-derived representation and one pseudo-compound representation, such as [[dam]+[age]]. These and other relevant distinctions related to combining forms (e.g., *thermo-* or -*plasm*) provide a set of items that can support novel questions and research related to the lexical representations of multimorphemic, pseudo-morphemic, and monomorphemic words.

Recognizing these potential affixes and combining forms within pseudo-compounds and other constructions may inform theoretical questions related to lexical access and the impact of morphological information (e.g., Rastle & Davis, 2008). At a macro-theory level, research with pseudo-compounds may aid in distinguishing between pre-lexical theories (e.g., Fiorentino & Poeppel, 2007; Taft & Forster, 1975, 1976), full-listing theories (e.g., Butterworth, 1983; Manelis & Tharp, 1977), post-lexical theories (Diependaele et al., 2005; Giraudo and Grainger 2000, 2001), dual-route theories (Baayen et al., 1997a; Diependaele et al., 2009), and distributed connectionist accounts (e.g., Baayen et al., 2011; Plaut & Gonnerman, 2000). Unlike the other groups of theories presented, distributed connectionist accounts conceptualize morphology as a learned set of word formation rules rather than discrete and symbolic units of meaning (Anderson, 1992; Plaut & Gonnerman, 2000). Each set of theories differs in their predictions of the relative influence and presence of orthographic, morphological, and semantic effects and the order or time-course of such effects. In brief consideration of the order of effects, recent research has highlighted the difficulty in evaluating and modeling the time-course of lexical processing, which further complicates the claims made by each set of theories (Leminen et al., 2019; Schmidtke et al., 2017; Schmidtke & Kuperman, 2019). To date, pseudo-compounds have been used to evaluate morphological effects predominantly in visual word recognition, and, more specifically, in masked priming experiments, so we focus on this literature to emphasize the benefit of word-word pseudo-compounds.

Word-word pseudo-compound constructions have been used within masked priming experiments to evaluate the availability of morphological representations in early stages of processing (e.g., Auch et al., 2023; Christianson et al., 2005; Gagné et al., 2018; Shoolman & Andrews, 2003). Different theories predict different outcomes of such an experiment. According to pre-lexical theory, the pseudo-morphemic representations of a pseudo-compound prime would become available and exert an influence on processing of the target because words are automatically decomposed into potential morphemes prior to lexical access (e.g., Rastle et al., 2004; Taft & Forster, 1975, 1976; Rastle & Davis, 2008). Full-listing and post-lexical approaches would predict that morphological information only becomes available after the full-word representation has been accessed, meaning that pseudo-morphemes do not become available to aid or hinder processing of the target (e.g., Manelis & Tharp, 1977). Dual-route theories might allow either outcome depending on the context of access and linguistic characteristics of the stimuli (e.g., Grainger & Ziegler, 2011; Schreuder & Baayen, 1995). Distributed connectionist models would make similar predictions to full-listing and post-lexical theories, but may be distinguished from these by allowing graded effects based on prior learning and the overall linguistic context as well as by using specific statistical methods to determine time-course of processing (Baayen et al., 2011; Jared et al., 2017; see Schmidtke et al., 2017, and Schmidtke & Kuperman, 2019, for further discussion and an example of using survival analysis to determine the order and timing of experimental effects). In sum, the use of word-word pseudo-compounds for informing psycholinguistic theories is still a relatively new, but promising, area of research (Auch et al., 2023; Chamberlain et al., 2020; Gagné et al., 2018).

Thus far, previous experiments have suggested that morphological information does become available for pseudo-compounds (i.e., for word-word pseudo-compounds such as *heathen*), even though such information is not part of the true morphemic structure. Experiments and theories differ, however, regarding the timing of this availability. The current database will facilitate the subsequent research needed to disentangle the various theoretical approaches that allow for morphological decomposition. For example, future research will be needed to systematically distinguish between connectionist/distributed semantic approaches, post-lexical theories, and dual-route approaches.

In addition to psycholinguistic research, LaDEP may be applicable to educational and clinical fields as either a resource for materials, or a means for investigating the effects of complexity and its different aspects on language learners and clinical populations. Educationally, knowledge of compounding and derivational morphology is related to language learning, reading, and writing success for both first and second language learners (Berko, 1958; Friedline, 2011; Kieffer & Lesaux, 2008; Kusumawardhani, 2018; Shum et al., 2016; Uygun & Gurel, 2017). To what extent, if any, does pseudo-morphological information prove to be a hindrance for language learners and early readers? Does the presence of pseudo-morphological information add complexity to the processing of this linguistic information? Clinically, morphological impairments can occur in acquired language disorders such as fluent and non-fluent aphasia (Dickey et al., 2008; Libben, 1990; Luzzatti et al., 2001; Nault, 2010; Semenza et al., 1997; Tyler & Cobb, 1987). Further, different aspects of those morphological constituents have been shown to impact processing for these individuals, which can be manipulated during therapy activities (e.g., Ciaccio et al., 2020; Nault, 2010). Are these impairments limited to true morphemes, or could they be influenced by the presence of pseudo-morphemes?

To conclude, the current project presented the Large Database of English Pseudo-Compounds, a resource of nearly 7500 English pseudo-compounds for researchers and others to select stimuli, find control items, and create experimental questions, hypotheses, and paradigms. The database provides a large set of items with varying characteristics, including length, positional family size, and the presence or absence of affixation, which can facilitate the creation of novel research. Moreover, there are existing research questions related to the use of morphological and orthographic information where applying the pseudo-compounds in LaDEP, which are existing English words, may be particularly informative (e.g., evidence for pre-lexical vs. post-lexical theories). Possible clinical and educational applications include the investigation of issues related to complexity; that is, whether pseudo-morphological constructions impact the processing in clinical or language learning populations. LaDEP can facilitate research on pseudo-compound constructions and extend the literature on both compound words and other pseudo-morphological constructions. Ultimately, LaDEP will support the stimuli selection and experiment creation of researchers who wish to investigate the impact of pseudo-morphological information on lexical processing and production.

## Data Availability

The dataset presented in this study is available in the University of Alberta Education and Research Archive (ERA), https://era.library.ualberta.ca and can be found via the search term LaDEP. This study was not preregistered.

## References

[CR1] Anderson SR (1992). A-morphous morphology (No. 62).

[CR2] Auch, L., Gagné, C. L., Spalding, T. L. (2023). Consequences of morpheme access during the comprehension and production of three types of pseudo-compounds. Manuscript submitted for publication.

[CR3] Baayen RH, Piepenbrock R, Gulikers L (1995). The CELEX lexical database (release 2).

[CR4] Baayen RH, Dijkstra T, Schreuder R (1997). Singulars and plurals in Dutch: Evidence for a parallel dual-route model. Journal of Memory and Language.

[CR5] Baayen RH, Lieber R, Schreuder R (1997). The morphological complexity of simplex nouns. Linguistics.

[CR6] Baayen RH, Milin P, Đurđević DF, Hendrix P, Marelli M (2011). An amorphous model for morphological processing in visual comprehension based on naive discriminative learning. Psychological Review.

[CR7] Balota DA, Yap MJ, Hutchison KA, Cortese MJ, Kessler B, Loftis B, Neely JH, Nelson DL, Simpson GB, Treiman R (2007). The English Lexicon Project. Behavior Research Methods.

[CR8] Barton JJS, Hanif HM, Eklinder Björnström L, Hills C (2014). The word-length effect in reading: A review. Cognitive Neuropsychology.

[CR9] Berko J (1958). The child’s learning of English morphology. Word.

[CR10] Bertram R, Hyönä J (2003). The length of a complex word modifies the role of morphological structure: Evidence from eye movements when short and long Finnish compounds. Journal of Memory and Language.

[CR11] Bolinger DL (1948). On defining the morpheme. Word.

[CR12] Bronk M, Zwitserlood P, Bölte J (2013). Manipulations of word frequency reveal differences in the processing of morphologically complex and simple words in German. Frontiers in Psychology.

[CR13] Brysbaert M, New B (2009). Moving beyond Kučera and Francis: A critical evaluation of current word frequency norms and the introduction of a new and improved word frequency measure for American English. Behavior Research Methods.

[CR14] Butterworth B, Butterworth B (1983). Lexical representation. Language Production.

[CR15] Carstairs-McCarthy A (2017). Introduction to English Morphology: words and their structure.

[CR16] Chamberlain JM, Gagné CL, Spalding TL, Lõo K (2020). Detecting spelling errors in compound and pseudocompound words. Journal of Experimental Psychology: Learning, Memory, and Cognition.

[CR17] Chang YN, Hsu CH, Tsai JL, Chen CL, Lee CY (2016). A psycholinguistic database for traditional Chinese character naming. Behavior Research Methods.

[CR18] Christianson K, Johnson RL, Rayner K (2005). Letter Transpositions Within and Across Morphemes. Journal of Experimental Psychology: Learning, Memory, and Cognition.

[CR19] Ciaccio LA, Burchert F, Semenza C (2020). Derivational morphology in agrammatic aphasia: A comparison between prefixed and suffixed words. Frontiers in Psychology.

[CR20] Cop U, Dirix N, Drieghe D, Duyck W (2017). Presenting GECO: An eyetracking corpus of monolingual and bilingual sentence reading. Behavior Research Methods.

[CR21] Cohen, J. (1992). A power primer. *Psychological Bulletin, 112*(1), 155–159. 10.1037/0033-2909.112.1.155 10.1037//0033-2909.112.1.155 19565683

[CR22] Creemers A, Davies AG, Wilder RJ, Tamminga M, Embick D (2020). Opacity, transparency, and morphological priming: A study of prefixed verbs in Dutch. Journal of Memory and Language.

[CR23] Crepaldi D, Rastle K, Davis CJ (2010). Morphemes in their place: Evidence for position-specific identification of suffixes. Memory & Cognition.

[CR24] Crepaldi D, Rastle K, Davis CJ, Lupker SJ (2013). Seeing stems everywhere: position-independent identification of stem morphemes. Journal of Experimental Psychology: Human Perception and Performance.

[CR25] De Jong IV NH, Schreuder R, Harald Baayen R (2000). The morphological family size effect and morphology. Language and Cognitive Processes.

[CR26] Dickey MW, Milman LH, Thompson CK (2008). Judgment of functional morphology in agrammatic aphasia. Journal of Neurolinguistics.

[CR27] Diependaele K, Sandra D, Grainger J (2005). Masked cross-modal morphological priming: Unravelling morpho-orthographic and morpho-semantic influences in early word recognition. Language and Cognitive Processes.

[CR28] Diependaele K, Sandra D, Grainger J (2009). Semantic transparency and masked morphological priming: The case of prefixed words. Memory & Cognition.

[CR29] Duñabeitia JA, Laka I, Perea M, Carreiras M (2009). Is Milkman a superhero like Batman? Constituent morphological priming in compound words. European Journal of Cognitive Psychology.

[CR30] Etymonline (2021). *Online Etymology Dictionary*. https://www.etymonline.com/

[CR31] Feldman LB, Pastizzo MJ (2003). Morphological facilitation: The role of semantic transparency and family size. Trends in Linguistics Studies and Monographs.

[CR32] Ferrand, L., Méot, A., Spinelli, E., New, B., Pallier, C., Bonin, P., ... & Grainger, J. (2018). MEGALEX: A megastudy of visual and auditory word recognition. *Behavior Research Methods*, *50*(3), 1285–1307. 10.3758/s13428-017-0943-1 28791657

[CR33] Fiorentino R, Fund-Reznicek E (2009). Masked morphological priming of compound constituents. The Mental Lexicon.

[CR34] Fiorentino R, Poeppel D (2007). Compound words and structure in the lexicon. Language and Cognitive Processes.

[CR35] Fradin B (2000). Combining forms, blends and related phenomena. Extragrammatical and Marginal Morphology.

[CR36] Friedline, B. E. (2011). Challenges in the second language acquisition of derivational morphology: From theory to practice (Publication No. 3485664). [Doctoral dissertation, University of Pittsburgh]. University of Pittsburgh ProQuest Dissertations Publishing.

[CR37] Gagné CL, Spalding TL (2016). Effects of morphology and semantic transparency on typing latencies in English compound and pseudocompound words. Journal of Experimental Psychology: Learning, Memory, and Cognition.

[CR38] Gagné CL, Spalding TL, Nisbet KA, Armstrong C (2018). Pseudo-morphemic structure inhibits, but morphemic structure facilitates, processing of a repeated free morpheme. Language, Cognition and Neuroscience.

[CR39] Gagné CL, Spalding TL, Schmidtke D (2019). LaDEC: The large database of English compounds. Behavior Research Methods.

[CR40] Giraudo H, Grainger J (2000). Effects of prime word frequency and cumulative root frequency in masked morphological priming. Language and Cognitive Processes.

[CR41] Giraudo H, Grainger J (2001). Priming complex words: Evidence for supralexical representation of morphology. Psychonomic Bulletin & Review.

[CR42] Grainger J, Ziegler JC (2011). A dual-route approach to orthographic processing. Frontiers in Psychology.

[CR43] Hanssen E, Banga A, Schreuder R, Neijt A (2013). Semantic and prosodic effects of Dutch linking elements. Morphology.

[CR44] Hyönä J, Olson RK (1995). Eye fixation patterns among dyslexic and normal readers: effects of word length and word frequency. Journal of Experimental Psychology: Learning, Memory, and Cognition.

[CR45] Iacobini C (1997). Distinguishing derivational prefixes from initial combining forms. Mediterranean Morphology Meetings.

[CR46] Inhoff AW (1989). Lexical access during eye fixations in reading: Are word access codes used to integrate lexical information across interword fixations?. Journal of Memory and Language.

[CR47] Jared D, Jouravlev O, Joanisse MF (2017). The effect of semantic transparency on the processing of morphologically derived words: Evidence from decision latencies and event-related potentials. Journal of Experimental Psychology: Learning, Memory, and Cognition.

[CR48] Juhasz BJ, Lai YH, Woodcock ML (2015). A database of 629 English compound words: ratings of familiarity, lexeme meaning dominance, semantic transparency, age of acquisition, imageability, and sensory experience. Behavior Research Methods.

[CR49] Juhasz, B. J. (2006). Effects of word length and sentence context on compound word recognition: An eye movement investigation. [Doctoral dissertation, University of Massachusetts Amherst]. https://scholarworks.umass.edu/dissertations/AAI3215917

[CR50] Keuleers E, Diependaele K, Brysbaert M (2010). Practice effects in large-scale visual word recognition studies: A lexical decision study on 14,000 Dutch mono-and disyllabic words and nonwords. Frontiers in Psychology.

[CR51] Keuleers E, Lacey P, Rastle K, Brysbaert M (2012). The British Lexicon Project: Lexical decision data for 28,730 monosyllabic and disyllabic English words. Behavior Research Methods.

[CR52] Kieffer MJ, Lesaux NK (2008). The role of derivational morphology in the reading comprehension of Spanish-speaking English language learners. Reading and Writing.

[CR53] Kim SY, Yap MJ, Goh WD (2018). The role of semantic transparency in visual word recognition of compound words: A megastudy approach. Behavior Research Methods.

[CR54] Kusumawardhani P (2018). The error analysis of derivational morphology in EFL’s english narrative composition. International Journal of Language Education.

[CR55] Lehrer A (1998). Scapes, holics, and thons: The semantics of English combining forms. American Speech.

[CR56] Leminen A, Smolka E, Duñabeitia JA, Pliatsikas C (2019). Morphological processing in the brain: The good (inflection), the bad (derivation) and the ugly (compounding). Cortex.

[CR57] Li, J., Bhattasali, S., Zhang, S., Franzluebbers, B., Luh, W. M., Spreng, R. N., ... & Hale, J. (2022). Le Petit Prince multilingual naturalistic fMRI corpus. *Scientific Data*, *9*(1), 1–15. 10.1038/s41597-022-01625-7 PMC9424229 36038567

[CR58] Libben G (1990). Morphological representations and morphological deficits in aphasia. Morphology, Phonology, and Aphasia.

[CR59] Lima SD, Pollatsek A (1983). Lexical access via an orthographic code? The Basic Orthographic Syllabic Structure (BOSS) reconsidered. Journal of Verbal Learning and Verbal Behavior.

[CR60] Luzzatti C, Mondini S, Semenza C (2001). Lexical representation and processing of morphologically complex words: Evidence from the reading performance of an Italian agrammatic patient. Brain and Language.

[CR61] MacGregor LJ, Shtyrov Y (2013). Multiple routes for compound word processing in the brain: Evidence from EEG. Brain and Language.

[CR62] Mailhot H, Wilson MA, Macoir J, Deacon SH, Sánchez-Gutiérrez C (2020). MorphoLex-FR: A derivational morphological database for 38,840 French words. Behavior Research Methods.

[CR63] Manelis L, Tharp DA (1977). The processing of affixed words. Memory & Cognition.

[CR64] Marelli M, Luzzatti C (2012). Frequency effects in the processing of Italian nominal compounds: Modulation of headedness and semantic transparency. Journal of Memory and Language.

[CR65] Marslen-Wilson WD, Bozic M, Randall B (2008). Early decomposition in visual word recognition: Dissociating morphology, form, and meaning. Language and Cognitive Processes.

[CR66] Monsell S, Ellis AW (1985). Repetition and the lexicon. Progress in the Psychology of Language.

[CR67] Nikolaev A, Ashaie S, Hallikainen M, Hänninen T, Higby E, Hyun J, Lehtonen M, Soininen H (2019). Effects of morphological family on word recognition in normal aging, mild cognitive impairment, and Alzheimer’s disease. Cortex.

[CR68] Nault, K. (2010). Morphological Therapy Protocol. [Doctoral dissertation, University of Alberta]. University of Alberta Education and Research Archive (ERA). 10.7939/R31Q6P

[CR69] Oxford University Press. (2021, December). Oxford English Dictionary Online. https://www.oed.com/

[CR70] Plaut DC, Gonnerman LM (2000). Are non-semantic morphological effects incompatible with a distributed connectionist approach to lexical processing?. Language and Cognitive Processes.

[CR71] Rastle K, Davis M (2008). Morphological decomposition based on the analysis of orthography. Language and Cognitive Processes.

[CR72] Rastle K, Davis MH, New B (2004). The broth in my brother’s brothel: Morpho-orthographic segmentation in visual word recognition. Psychonomic Bulletin & Review.

[CR73] Sánchez-Gutiérrez CH, Mailhot H, Deacon SH, Wilson MA (2018). MorphoLex: A derivational morphological database for 70,000 English words. Behavior Research Methods.

[CR74] Sandra D (1990). On the Representation and Processing of Compound Words: Automatic Access to Constituent Morphemes Does Not Occur. The Quarterly Journal of Experimental Psychology Section A.

[CR75] Schmidtke D, Kuperman V (2019). A paradox of apparent brainless behavior: The time-course of compound word recognition. Cortex.

[CR76] Schmidtke D, Matsuki K, Kuperman V (2017). Surviving blind decomposition: A distributional analysis of the time-course of complex word recognition. Journal of Experimental Psychology: Learning, Memory, and Cognition.

[CR77] Schmidtke D, Van Dyke JA, Kuperman V (2021). CompLex: An eye-movement database of compound word reading in English. Behavior Research Methods.

[CR78] Schreuder R, Baayen RH (1995). Modeling morphological processing. Morphological Aspects of Language Processing.

[CR79] Schreuder R, Baayen RH (1997). How complex simplex words can be. Journal of Memory and Language.

[CR80] Semenza C, Luzzatti C, Carabelli S (1997). Morphological representation of compound nouns: A study on Italian aphasic patients. Journal of Neurolinguistics.

[CR81] Shillcock R (1990). Lexical Hypotheses in Continuous Speech. Cognitive Models of Speech Processing.

[CR82] Shoolman N, Andrews S, Kinoshita S, Lupker SJ (2003). Racehorses, reindeer, and sparrows: Using masked priming to investigate morphological influences on compound word identification. Masked priming: The state of the art.

[CR83] Shum KK, Ho CS, Siegel LS, Au TK (2016). First-language longitudinal predictors of second-language literacy in young L2 learners. Reading Research Quarterly.

[CR84] Siegelman, N., Schroeder, S., Acartürk, C., Ahn, H. D., Alexeeva, S., Amenta, S., ... & Kuperman, V. (2022). Expanding horizons of cross-linguistic research on reading: The Multilingual Eye-movement Corpus (MECO). *Behavior Research Methods, 54*, 2843–2863. 10.3758/s13428-021-01772-6 PMC8809631 35112286

[CR85] StataCorp (2019). Stata Statistical Software: Release 16.

[CR86] Stites MC, Federmeier KD, Christianson K (2016). Do morphemes matter when reading compound words with transposed letters? Evidence from eye-tracking and event-related potentials. Language, Cognition and Neuroscience.

[CR87] Taft M (1981). Prefix stripping revisited. Journal of Verbal Learning and Verbal Behavior.

[CR88] Taft M, Forster KI (1975). Lexical storage and retrieval of prefixed words. Journal of Verbal Learning and Verbal Behavior.

[CR89] Taft M, Forster KI (1976). Lexical storage and retrieval of polymorphemic and polysyllabic words. Journal of Verbal Learning and Verbal Behavior.

[CR90] The Center for Reading Research (2023). Word megastudy data and eye movement corpora available.

[CR91] Tse CS, Yap MJ, Chan YL, Sze WP, Shaoul C, Lin D (2017). The Chinese Lexicon Project: A megastudy of lexical decision performance for 25,000+ traditional Chinese two-character compound words. Behavior Research Methods.

[CR92] Tyler LK, Cobb H (1987). Processing bound grammatical morphemes in context: The case of an aphasic patient. Language and Cognitive Processes.

[CR93] Uygun S, Gürel A (2017). Compound processing in second language acquisition of English. Journal of the European Second Language Association.

[CR94] Whiting CM, Marslen-Wilson WD, Shtyrov Y (2013). Neural dynamics of inflectional and derivational processing in spoken word comprehension: laterality and automaticity. Frontiers in Human Neuroscience.

[CR95] Wolfram Research Inc., (2019). Mathematica (12.0.0). Champaign, USA: Wolfram Research.

